# From Contact Screening to Preventive Treatment Provision; Progress and Bottlenecks in High Tuberculosis Burden Settings

**DOI:** 10.51894/001c.160047

**Published:** 2026-04-08

**Authors:** Sobia Faisal, Sana Durvesh, Hassan Mahmood, Syed AzizurRab, Khalid Farough, Raja Farrukh, Furqan Irfan

**Affiliations:** 1 TB Project Greenstar Social Marketing Pakistan; 2 Greenstar Social Marketing Pakistan; 3 Integral Global; 4 Chief Executive Officer Greenstar Social Marketing Pakistan; 5 Department of Neurology and Ophthalmology, College of Osteopathic Medicine, Institute of Global Health Michigan State University

**Keywords:** Contact Tracing, Tuberculosis Preventive Treatment, Tuberculosis, Household Contacts

## Abstract

**Background:**

Tuberculosis (TB) preventive treatment (TPT) remains poorly utilized (5.4%) among eligible household contacts in Pakistan despite programmatic expansion. A critical programmatic knowledge gap exists regarding the magnitude and distribution of pre-initiation attrition among household (HH) contacts, including which stages of the care cascade and health facility types at which losses occur in Pakistan. This study aimed to quantify attrition of HH contacts of bacteriologically positive (B-pos) TB patients before TPT initiation at different stages of the care cascade.

**Methods:**

This retrospective cohort evaluated programmatic TB data from HH contacts of B-pos pulmonary TB patients managed through Public-Private Mix (PPM) facilities in 28 districts of Pakistan from January-July 2025. Contacts were verbally screened, assessed using chest radiography to exclude TB disease, and eligible individuals were offered TPT.

**Results:**

Among 20,290 index TB patients, 77,771 HH contacts were identified. Of all identified HH contacts, 55% (42,410/77,771) underwent verbal screening. Among those screened, 32% (13,395/42,410) proceeded to chest X-ray screening and 98% (13,145/13,395) started TPT. Around 4% (1,505) were identified as presumptive TB cases and 52% (792/1,505) were diagnosed with TB. Substantial pre-treatment attrition occurred at two critical stages: 45% prior to verbal screening and 63% prior to X-ray screening. Children aged 0–4 years had the lowest TPT initiation (13.1%), despite being a high-risk group.

**Conclusion:**

Substantial losses before TPT initiation occur during contact screening and disease exclusion stages, indicating programmatic challenges requiring targeted operational strategies. Only 16.9% of household contacts completed the care cascade, highlighting a major missed opportunity for TB prevention.

## INTRODUCTION

Approximately one-quarter of the global population is infected with tuberculosis (TB), and 5–10% will develop active disease during their lifetime. The United Nations High-Level Meeting (UNHLM) on TB has set targets to treat at least 90% of individuals at high risk of developing TB.^1^ As a high TB-burden country, Pakistan continues to face significant public health challenges related to TB incidence.^2^

To prevent progression from infection to active disease, the World Health Organization (WHO) recommends TB preventive treatment (TPT) for high-risk groups, including household contacts of bacteriologically positive pulmonary TB patients, people living with HIV, and other vulnerable populations.^3^ Pakistan adopted the WHO End TB Strategy as national policy in 2016 and subsequently developed and implemented national TPT guidelines targeting these high-risk groups.^4^ Despite these efforts, only 5.4% of the eligible household contacts of B-pos TB patients received TPT during 2024.^2^

Programmatic implementation of TPT involves multiple stakeholders including TB program managers, healthcare providers and affected communities.^3^ Effective implementation requires a clear policy framework for TB control, strong leadership, coordination across programs, and an uninterrupted supply of medications, supported by robust surveillance systems and adequate access to laboratory and radiological services.^5,6^ Healthcare personnel must be adequately engaged and confident in the effectiveness of TPT as a strategy to reduce TB transmission.^7,8^ In addition, patients and their families should be educated about TPT to improve uptake and adherence.^9^

Global evidence indicates that, despite the inclusion of TPT services in national protocols, effective implementation remains limited.^5^ To inform evidence-based programmatic improvements, this study evaluates attrition across different stages of the TPT cascade and across service delivery models among household contacts of TB patients in Pakistan. This study aimed to evaluate attrition across the TPT cascade, compare uptake across service delivery models, and examine TB case identification among household contacts.

## METHODS

### Study Design

This study was an operational evaluation using a retrospective cohort design based on routine programmatic data. It analyzed TPT program data implemented through the Public–Private Mix (PPM) model of Greenstar Social Marketing (GSM) in Pakistan. The process began with identification of bacteriologically positive pulmonary TB patients, followed by household contact tracing, exclusion of active TB disease, and provision of TPT services to eligible individuals.

### Study Settings

The study included data on eligible household contacts of patients who initiated TB treatment between January and July 2025 across 28 Public–Private Mix (PPM) implementation districts in Pakistan. Within the PPM model, TB care services were delivered through four platforms: PPM 1—private general practitioners (GPs) operating community-based clinics; PPM 2—health facilities, primarily hospitals, managed by non-governmental organizations (NGOs); PPM 3—large private hospitals (LPHs) with onsite specialized services; and PPM 4—parastatal hospitals.^4^

**Figure 1.**
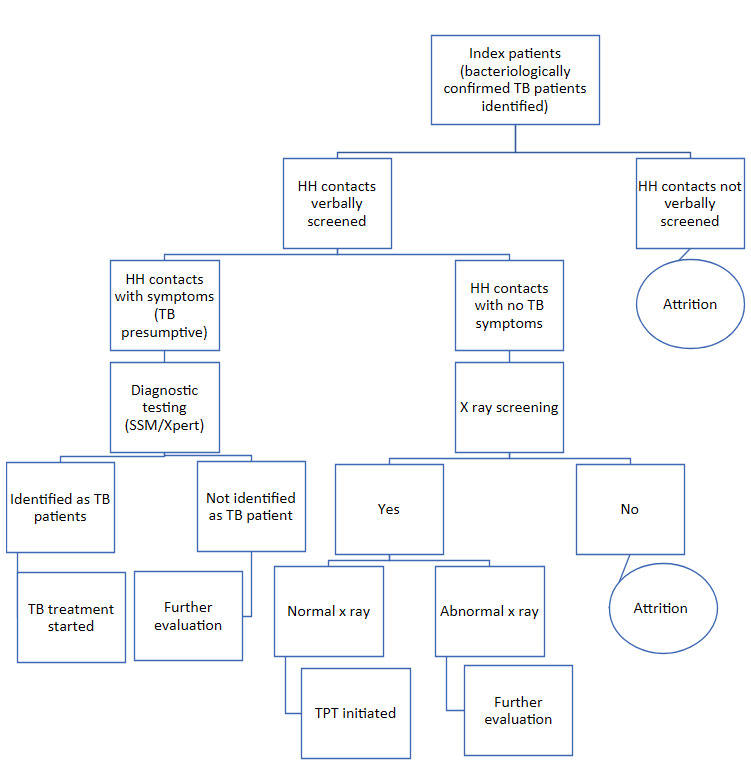
Attrition of HH contacts of TB patients in care cascade

Household contacts of pulmonary TB patients were screened for TB symptoms. Asymptomatic contacts were referred for chest X-ray screening to exclude active TB disease, and those eligible were offered TPT (Figure 1). A household contact was defined as an individual who shared one or more nights with the index patient during the three months preceding TB diagnosis.^10^

To facilitate access, the program provided financial support to household contacts, including transportation reimbursement (PKR 600 / ~$2 USD per contact), free chest X-ray services (vendor reimbursement of approximately PKR 800 / ~$3 USD per contact), and coverage of doctor consultation fees (PKR 400 / ~$1.4 USD per contact). The contact screening process varied slightly across PPM models. In the GP-based model (PPM 1), field staff visited the household of the index patient to conduct verbal screening. In hospital-based models (PPM 2–4), household contacts were typically contacted by phone and requested to visit the facility for evaluation, although home visits were conducted when necessary. Household contacts without symptoms and without contraindications (e.g., liver disease, pregnancy) were referred for chest X-ray screening. In the GP model, contacts were directed to nearby networked X-ray facilities, whereas in hospital-based models, X-ray screening was conducted onsite or at affiliated facilities depending on service availability.

### Study Population

The study population comprised household contacts of bacteriologically positive (B-pos) pulmonary TB patients registered within the program at Public–Private Mix (PPM) sites.

All individuals identified as household contacts of B-pos pulmonary TB patients during the study period were included. Contacts with incomplete records or those not meeting the household contact definition (i.e., not residing with the index patient during the specified time frame) were excluded during contact investigation. Incomplete records were defined as those lacking key cascade variables, such as contact identification or screening status/results, and were excluded during data validation prior to dataset cleaning.

A universal sampling approach was employed, whereby all eligible household contacts were included in the analysis. Data were initially recorded in patient record sheets by program implementation teams and subsequently entered into an electronic Management Information System (MIS) by trained personnel.

The primary outcome (dependent variable) was TPT initiation. Independent variables included age group, service delivery model, geographic distribution, screening and diagnostic processes, and time period.

Data analysis was performed using MedCalc statistical software (version 23.2).11 Analysis was limited to bivariate comparisons, as the use of aggregated programmatic data did not allow for reliable adjustment for potential confounders in multivariable models. Associations between categorical variables were assessed using the Chi-square test, with a p-value < 0.05 considered statistically significant.

TPT cascade analysis was conducted to assess progression across sequential stages of care. Among identified contacts, a proportion underwent verbal screening; of those screened, some were classified as presumptive TB and referred for diagnostic evaluation, while the remainder were asymptomatic. Asymptomatic contacts were further assessed for contraindications, and eligible individuals were referred for chest X-ray screening. Among those screened by X-ray, a subset initiated TPT. At each stage, the proportion progressing to the subsequent step was calculated.

Eligibility for TPT included individuals who were asymptomatic on verbal screening, had no evidence of TB disease on chest X-ray (i.e., normal or not suggestive of TB), and had no contraindications to treatment (Figure 1).

Ethical approval for the study was obtained from National Iqra University, Pakistan (Approval No. INU/ERC/24-1). The requirement for informed consent was waived, as the study utilized anonymized secondary programmatic data without direct patient interaction.

## RESULTS

During the study period, 20,290 bacteriologically positive (B-pos) pulmonary TB patients were identified, with a total of 77,771 household contacts (mean 3.83 contacts per index case). These patients were registered across different service delivery models: 67.9% (13,783/20,290) in the general practitioner (GP) model, 28.8% (5,840/20,290) in large private hospitals, 1.6% (330/20,290) in NGO-led hospitals, and 1.7% (337/20,290) in parastatal hospitals (Table 1).

**Table 1.** From contact tracing till TPT initiation.

**Model**	*B-pos pulmonary TB patients	**HH contacts	**Verbally screened**		**X-ray screened among verbally screened**		**HH started TPT**	***TPT started among **HH contacts**
**Service delivery**	n		n	%	N	%	N	%
General Physician Model	13783	51494	32048	62%	7019	22%	6835	13.27%
Large Private Hospital	5840	23637	9408	40%	5736	61%	5719	24.20%
Non-Governmental Organizations Led Hospital	330	1187	527	44%	331	63%	305	25.70%
Parastatal Hospital	337	1453	427	29%	309	72%	286	19.68%
Total	20290	77771	42410	55%	13395	32%	13145	16.90%
**Time Trend**								
Jan-25	2637	10345	5657	55%	1587	28%	1564	15.12%
Feb-25	2812	11152	5985	54%	1722	29%	1721	15.43%
Mar-25	3096	11558	6339	55%	1927	30%	1896	16.40%
Apr-25	2908	11418	6333	55%	1995	32%	1845	16.16%
May-25	3133	11707	6557	56%	2506	38%	2501	21.36%
Jun-25	2824	10400	5678	55%	1894	33%	1870	17.98%
Jul-25	2880	11191	5861	52%	1764	30%	1748	15.62%
Total	20290	77771	42410	55%	13395	32%	13145	16.90%
**Age in years**								
0-4		4925	2533	51%	742	29%	647	13.14%
5-14.		15510	7868	51%	2560	33%	2493	16.07%
15 plus		57336	32009	56%	10093	32%	10005	17.45%
Total		77771	42410	55%	13395	32%	13145	16.90%

*B-pos: bacteriologically confirmed

**HH: household contacts

***TPT: TB preventive treatment

Among the household contacts, 6.3% (4,925/77,771) were aged 0–4 years, 19.9% (15,510/77,771) were aged 5–14 years, and 73.7% (57,336/77,771) were aged ≥15 years.

Verbal screening was conducted for 55% (42,410/77,771) of all household contacts, of whom 4% (1,505/42,410) were identified as presumptive TB cases. Among asymptomatic contacts without contraindications to TPT, 32% (13,395/42,410) underwent chest X-ray screening. Of those screened, 98% (13,145/13,395) initiated TPT (Table 1).

### Model-wise Comparison

The GP model, which accounted for the largest proportion of patients and household contacts, achieved the highest verbal screening coverage at 62% (32,048/51,494), followed by NGO-led hospitals at 44% (527/1,187), large private hospitals at 40% (9,408/23,637), and parastatal hospitals at 29% (427/1,453).

In contrast, the proportion of household contacts who reached health facilities for chest X-ray screening was highest in parastatal hospitals at 72% (309/427), followed by NGO-led hospitals at 63% (331/527), large private hospitals at 61% (5,736/9,408), and lowest in the GP model at 22% (7,019/32,048).

TPT initiation among household contacts was highest in NGO-led hospitals at 25.7% (305/1,187), followed by large private hospitals at 24.2% (5,719/23,637), parastatal hospitals at 19.68% (286/1,453), and lowest in the GP model at 13.27% (6,835/51,494) (Table 1). There was a statistically significant association between TPT initiation and the model of service delivery (p < 0.001).

### Time Trend

Over the study period, verbal screening coverage remained relatively stable, ranging from 52% to 56%. In contrast, chest X-ray screening increased from 28% in January to a peak of 38% in May 2025, before declining to 33% and 30% in June and July, respectively. Similarly, TPT initiation rose from 15.1% in January to 21.4% in May, followed by a decline to 18.0% and 15.6% in June and July 2025 (Table 1). These monthly differences were statistically significant (p < 0.001) and were assessed using chi-square tests of categorical proportions rather than formal trend regression analysis.

### Age-wise Comparison

TPT initiation varied across age groups, with the lowest uptake observed among children aged 0–4 years at 13.1% (647/4,925). Uptake increased to 16.7% (2,493/15,510) among those aged 5–14 years and was highest among individuals aged ≥15 years at 17.5% (10,005/57,336). Age was significantly associated with TPT initiation (p < 0.001). These findings are descriptive, as multivariable adjustment was not feasible due to the use of aggregated programmatic data.

### TB Disease Identification

During contact tracing, 4% (1,505/42,410) of verbally screened household contacts were identified as presumptive TB cases. This proportion varied across service delivery models, with the lowest observed in the GP model (3%) and higher proportions (6–7%) reported in hospital-based settings.

Confirmed TB disease among household contacts also differed by service delivery model, with the highest detection rate in NGO-led hospitals (2.53%), followed by large private hospitals (1.05%), parastatal hospitals (1.03%), and the lowest in the GP model (0.97%).

Age-wise, TB detection was highest among children aged 0–4 years (1.22%), followed by individuals aged ≥15 years (1.02%), and lowest among those aged 5–14 years (0.54%).

Overall, TB case identification among household contacts was significantly associated with both the model of care and age group (p < 0.001).

## DISCUSSION

This study demonstrated that only 16.9% (13,145/77,771) of identified household contacts initiated TPT, highlighting substantial attrition along the care cascade. The greatest losses occurred during the verbal screening stage, followed by the X-ray screening stage.

Pakistan, a high TB-burden lower-middle-income country (LMIC), has shown modest improvement in TPT uptake among eligible household contacts, increasing from 3.2% in 2023 to 5.4% in 2024.^2,11^ In this operational evaluation of the PPM model, a comparatively higher proportion (16.9%) of household contacts initiated TPT in 2025. However, this remains far below global targets of reaching at least 90% of individuals at risk.^1^

### Major Bottleneck 1: Verbal Screening Gap (45% attrition)

In this study, nearly half of household contacts (45%) were not reached for verbal screening, representing a major early loss in the care cascade. Effective contact tracing depends on several prerequisites, including patient consent to evaluate household members, residence within the facility catchment area for home-based screening, willingness of contacts to visit health facilities for evaluation, and availability of contacts at the time of tracing.

Barriers to contact tracing were observed at multiple levels—patient, provider, and health system. Patient-level barriers included cultural sensitivities, limited understanding of TB, mistrust in healthcare services, and low perceived risk among contacts. Provider-level challenges comprised inadequate training on TPT, difficulty locating contacts during visits, limited time for counselling, competing responsibilities, and resource constraints. Health system barriers included insufficient programmatic support^12–16^ and intermittent stockouts of TPT medications.^5^

### Major bottleneck 2: X-ray screening gap (63% attrition)

Attrition in the care cascade occurred not only at the verbal screening stage but also at the pre-treatment diagnostic stage, where a substantial proportion of asymptomatic household contacts did not complete chest X-ray screening. Accessing X-ray services often required travel to designated facilities, which imposed time, financial, and opportunity costs, including absence from work or school, as well as the need for sustained motivation. Long travel distances to health facilities remain a well-recognized barrier in contact tracing.^17^

During this program, financial support—including transportation reimbursement, free X-ray services, and consultation coverage—was provided to household contacts through Global Fund support. Despite these measures, overall TPT initiation remained low at 16.9%, indicating that financial barriers alone do not fully explain losses at this stage.

While attrition at the verbal screening stage primarily reflects challenges in access and engagement, losses at the X-ray stage are more indicative of logistical and diagnostic pathway constraints.

Age-related disparities further influenced TPT uptake.^18^ Children aged 0–4 years had the lowest initiation rates, followed by those aged 5–14 years, while the highest uptake was observed among individuals aged ≥15 years. These findings suggest that younger children—despite being at greater risk of disease progression—are less likely to access preventive therapy, whereas adults benefit more from existing screening and treatment pathways. Programmatic experience indicates that the availability of shorter, user-friendly regimens such as 3HP may facilitate higher uptake among adults, while adherence to longer regimens such as 6H remains challenging.

In our study, the X-ray services were availed by more than two times among patients seeking care from hospital setting compared to GP settings where community was accessing healthcare from neighborhood clinic. X-ray screening at community level may help in increasing the access of patients seeking care at GP model by offering proximity convenience.^19,20^

### Successes: High TPT uptake once X-ray is completed

The high rate of TPT initiation among individuals who completed chest X-ray screening suggests that those who progressed to this stage were already motivated and willing to initiate treatment, indicating a degree of self-selection within the cascade.

Programmatic observations from field implementation teams—although not formally captured in the quantitative dataset—suggest that intermittent stockouts of TPT medications in certain districts may have contributed to delays in X-ray screening. These insights should be interpreted cautiously, as they were not derived from systematically collected data.

### Key Points

Notably, 45% of identified household contacts were not verbally screened for TB symptoms, representing a substantial initial loss in the cascade. Furthermore, among asymptomatic contacts potentially eligible for TPT, 63% did not complete chest X-ray screening, highlighting a critical point of attrition prior to treatment initiation.

**Figure 2.**
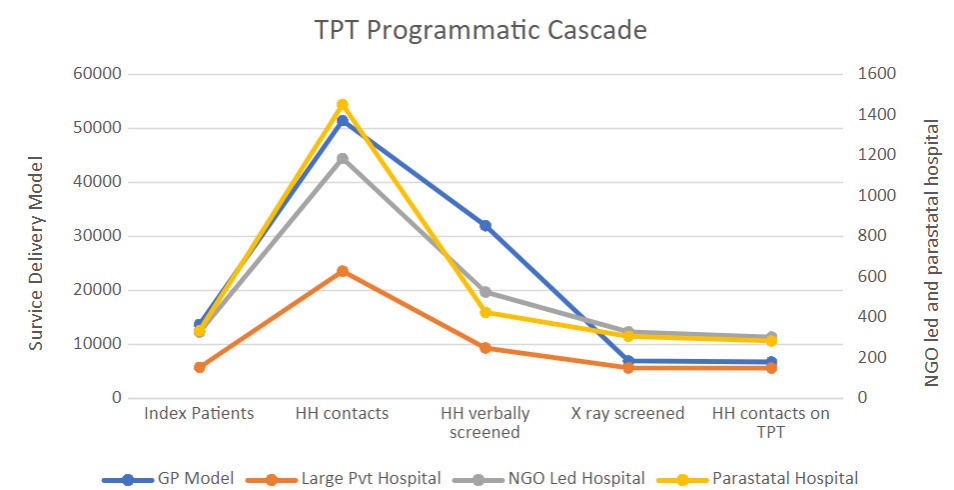
Attrition of HH contacts of TB patients in different health service delivery models.

The GP model contributed the largest number of bacteriologically confirmed TB patients and achieved the highest coverage of verbal contact screening. However, it also had the lowest proportion of household contacts progressing to X-ray screening, indicating a key bottleneck in accessing diagnostic services. In contrast, hospital-based models demonstrated better access to X-ray services (Figure 2).

Importantly, TPT uptake was high among household contacts who completed X-ray screening, reinforcing that the primary barriers lie in reaching and completing earlier stages of the cascade rather than in treatment acceptance.

## LIMITATIONS

This study has several limitations. The use of routine programmatic data limited control over data completeness and quality. Potential misclassification or under-documentation within the Management Information System (MIS) may have influenced the accuracy of cascade estimates. The findings may have limited generalizability beyond Public–Private Mix (PPM) settings. Additionally, the absence of qualitative data restricted the ability to validate and fully understand the underlying reasons for observed cascade losses.

## CONCLUSION

This study demonstrates substantial attrition in household contact management prior to TPT initiation, particularly during contact tracing and disease exclusion stages. The magnitude of attrition varied across healthcare delivery models, underscoring the need for model-specific programmatic strategies.

Key programmatic priorities include decentralizing access to screening services, strengthening contact tracing mechanisms, and enhancing counselling support. These recommendations are based on observed gaps in the care cascade and should be interpreted as operational implications rather than causal inferences.

To effectively scale up TPT, both demand- and supply-side barriers must be addressed. Demand-side challenges include limited awareness of TPT benefits and persistent stigma, while supply-side constraints involve the availability of TPT medications and adequately trained healthcare providers.

Targeted educational and counselling interventions for household contacts and affected communities should be prioritized to improve acceptance, uptake, and adherence to TPT.

### CONFLICT OF INTEREST

The authors confirm that the research was conducted independently without any conflict of interest that can affect results or their interpretations.
